# Supplementary material for: Image‐based deep learning reveals the responses of human motor neurons to stress and VCP‐related ALS
Source: Neuropathol Appl Neurobiol. 2021 Oct 18;48(2):e12770. doi: 10.1111/nan.12770 (PMC9298273; doi:10.1111/nan.12770)
Supplement: Supplementary file 1 — Figure S1. A Motor neuron characterisation. Representative images of iPSC‐derived motor neurons, immunolabeled with motor neuron specific markers SMI‐32 and ChAT. Scale bar = 10 μm. B. Schematic depicting the CNN‐based approach. Pre‐processing: [1] 16‐bit z‐stack raw images are merged using the Maximum Intensity Projection (MIP); [2] the MIP images are then converted to 8‐bit images and the different channels are merged together, depending on the protocol (either 1, 2 or 3 channels); [3] contrast is enhanced with a cut‐off of 0.1%; [4] each image (size = 1080x1080 pixels) is divided into 16 images of 270x270 pixels; (5) images are then resized into 224x224 pixels; (6) images are normalized the same way as the ImageNet dataset, using mean = [0.485, 0.456, 0.406] and std = [0.229, 0.224, 0.225]. Training: Concept illustration of training a neural network for binary classification of one condition against another. The train set is composed of labelled images for both conditions and is used to train the network, which assigns more weight to discriminating features in the images. Performance Evaluation: A distinct set of labelled images, the test set, is used to evaluate the performance of the trained network using metrics such as the Area Under the receiver operating characteristic Curve (AUC). The performances of different classifiers trained with different markers can be compared to uncover the relevance of specific markers in discriminating two conditions. Prediction: The trained model can then evaluate the probability for a given unlabelled image to belong to either one or the other condition. Figure S2. (A) Boxplots showing the distributions of model performances as evaluated using the AUC for classifiers trained using DAPI or ALS‐related RBP markers to discriminate untreated from stressed MN cultures. Each classifier was submitted to 10‐fold cross‐validation in 5 different subsets of the data, resulting in 50 points per classifier. Boxplots display the five number [file NAN-48-0-s001.docx]

**SUPPLEMENTARY MATERIAL**

**Image-based deep learning reveals the responses of human motor neurons to stress and *VCP*-related ALS**

*Colombine Verzat^#^, Jasmine Harley^#^, Rickie Patani*, Raphaëlle Luisier**


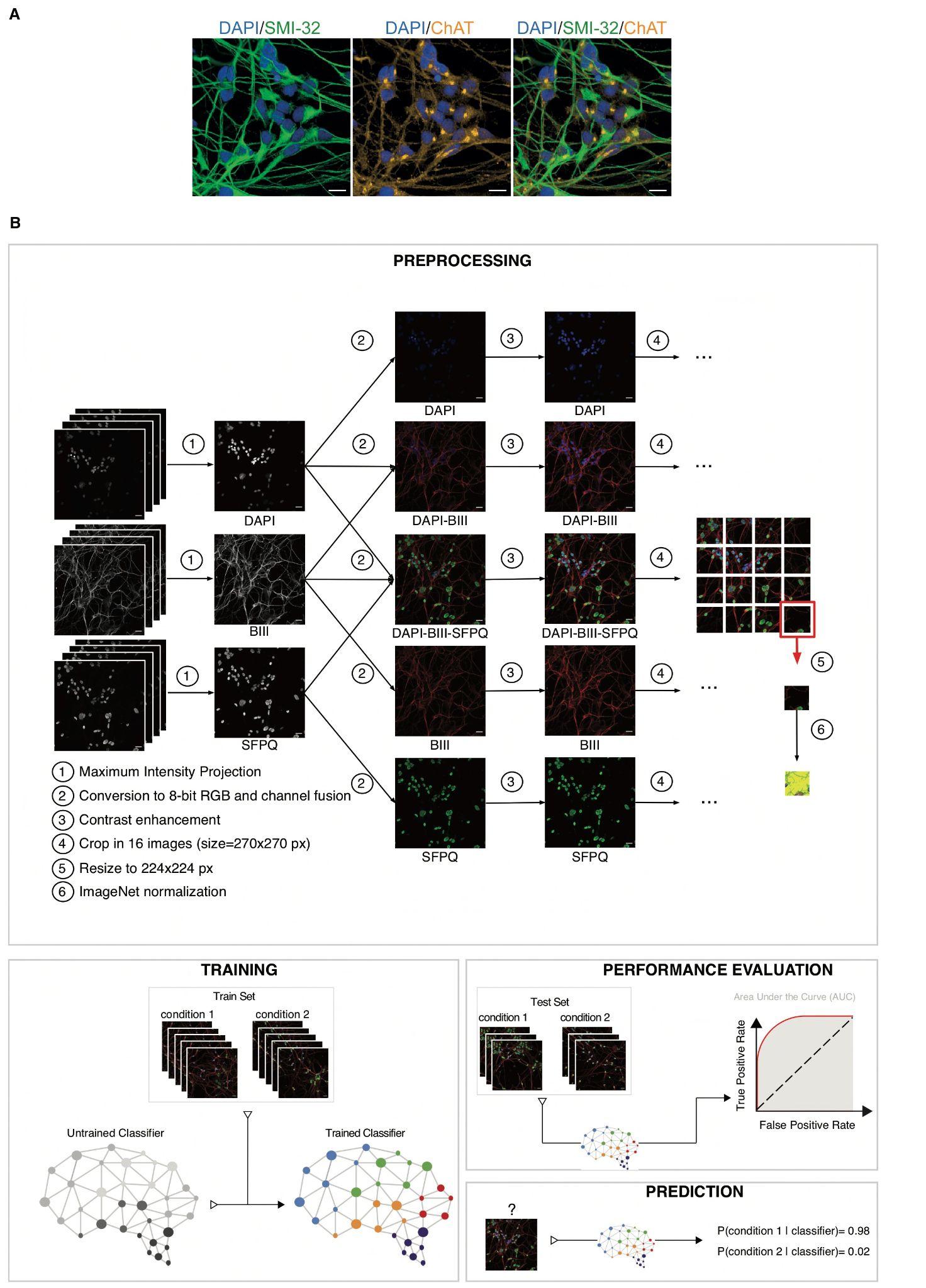


**Supplementary Figure 1 |**  **A.** Motor neuron characterisation. Representative images of iPSC-derived motor neurons, immunolabeled with motor neuron specific markers SMI-32 and ChAT. Scale bar = 10μm. **B.** Schematic depicting the CNN-based approach. **Pre-processing:** (1) 16-bit z-stack raw images are merged using the Maximum Intensity Projection (MIP); (2) the MIP images are then converted to 8-bit images and the different channels are merged together, depending on the protocol (either 1, 2 or 3 channels); (3) contrast is enhanced with a cut-off of 0.1%; (4) each image (size=1080x1080 pixels) is divided into 16 images of 270x270 pixels; (5) images are then resized into 224x224 pixels; (6) images are normalized the same way as the ImageNet dataset, using mean = [0.485, 0.456, 0.406] and std = [0.229, 0.224, 0.225]. **Training:** Concept illustration of training a neural network for binary classification of one condition against another. The train set is composed of labelled images for both conditions and is used to train the network, which assigns more weight to discriminating features in the images. **Performance Evaluation:** A distinct set of labelled images, the test set, is used to evaluate the performance of the trained network using metrics such as the Area Under the receiver operating characteristic Curve (AUC). The performances of different classifiers trained with different markers can be compared to uncover the relevance of specific markers in discriminating two conditions. **Prediction:** The trained model can then evaluate the probability for a given unlabelled image to belong to either one or the other condition.


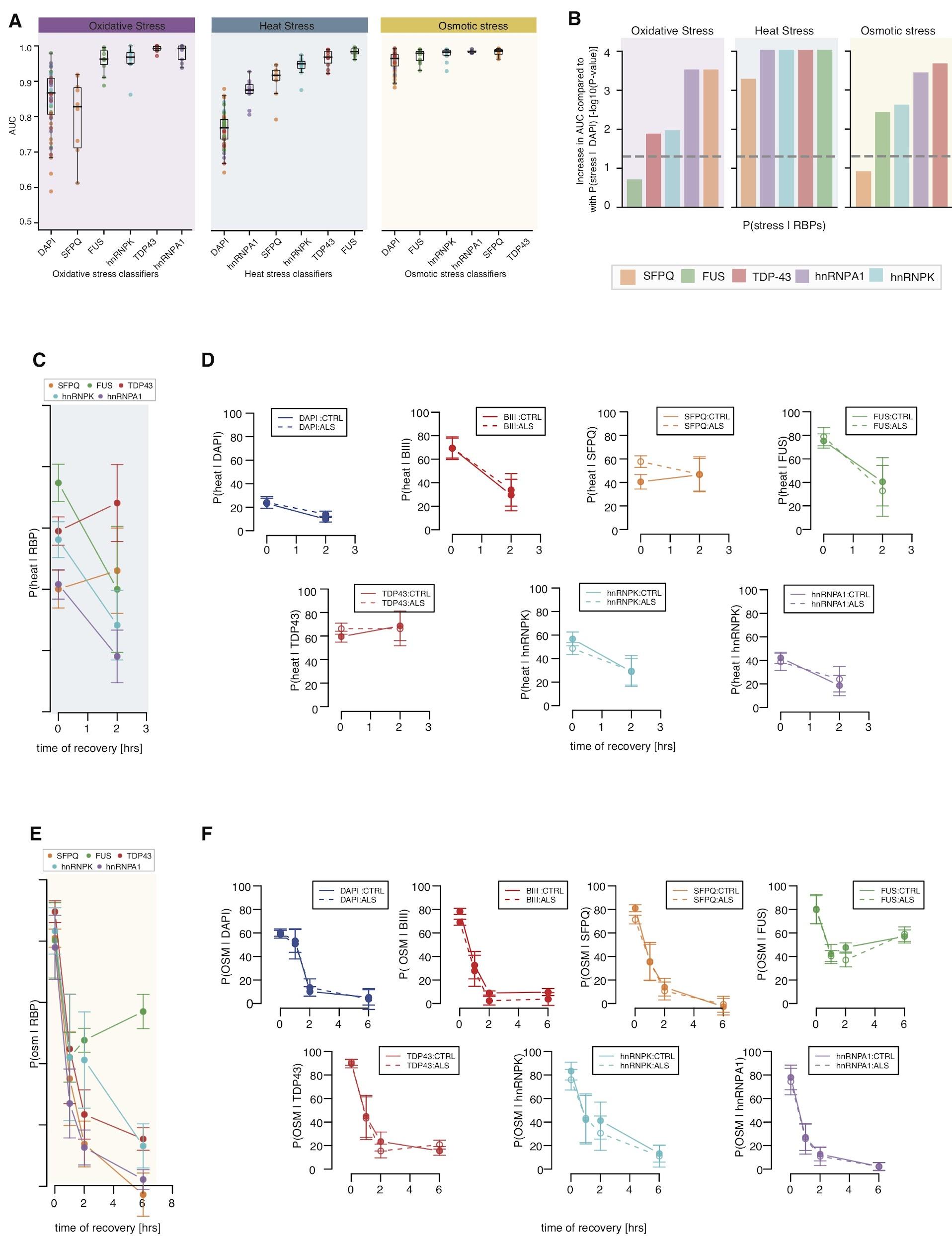


**Supplementary Figure 2 |** (**A**) Boxplots showing the distributions of model performances as evaluated using the AUC for classifiers trained using DAPI or ALS-related RBP markers to discriminate untreated from stressed MN cultures. Each classifier was submitted to 10-fold cross-validation in 5 different subsets of the data, resulting in 50 points per classifier. Boxplots display the five number summary of median, lower and upper quartiles, minimum and maximum values. P-values obtained from a one-sided Mann-Whitney test. (**B**) Bar graphs representing the increase in performance as obtained from -log10(P-values) of one-sided Mann-Whitney test comparing the AUCs from the *stress|DAPI* classifier and the AUCs from individual *stress|DAPI:RBP* classifiers for oxidative, heat and osmotic stresses. (**C**) Effect size (mean $\pm$ standard errors) of *heat|RBPs* classifier predictions of control MN cultures one hour after heat stress and two hours after recovery from heat stress. Effect size of the treatment at each time-point is obtained using linear mixed effects analysis accounting for idiosyncratic variations due to cell lines and experiment bias. (**D**) Same as (C) for individual *heat|RBPs* classifiers predictions. Solid lines = control MN cultures. Dashed lines = VCP-mutant MN cultures. (**E**) Effect size (mean $\pm$ standard errors) of *osm|RBPs* classifiers predict control MN cultures one hour after osmotic stress and one, two and six hours after recovery from osmotic stress. (**F**) Same as (E) for individual *osm|RBPs* classifiers predictions. Solid lines = control MN cultures. Dashed lines = VCP-mutant MN cultures.


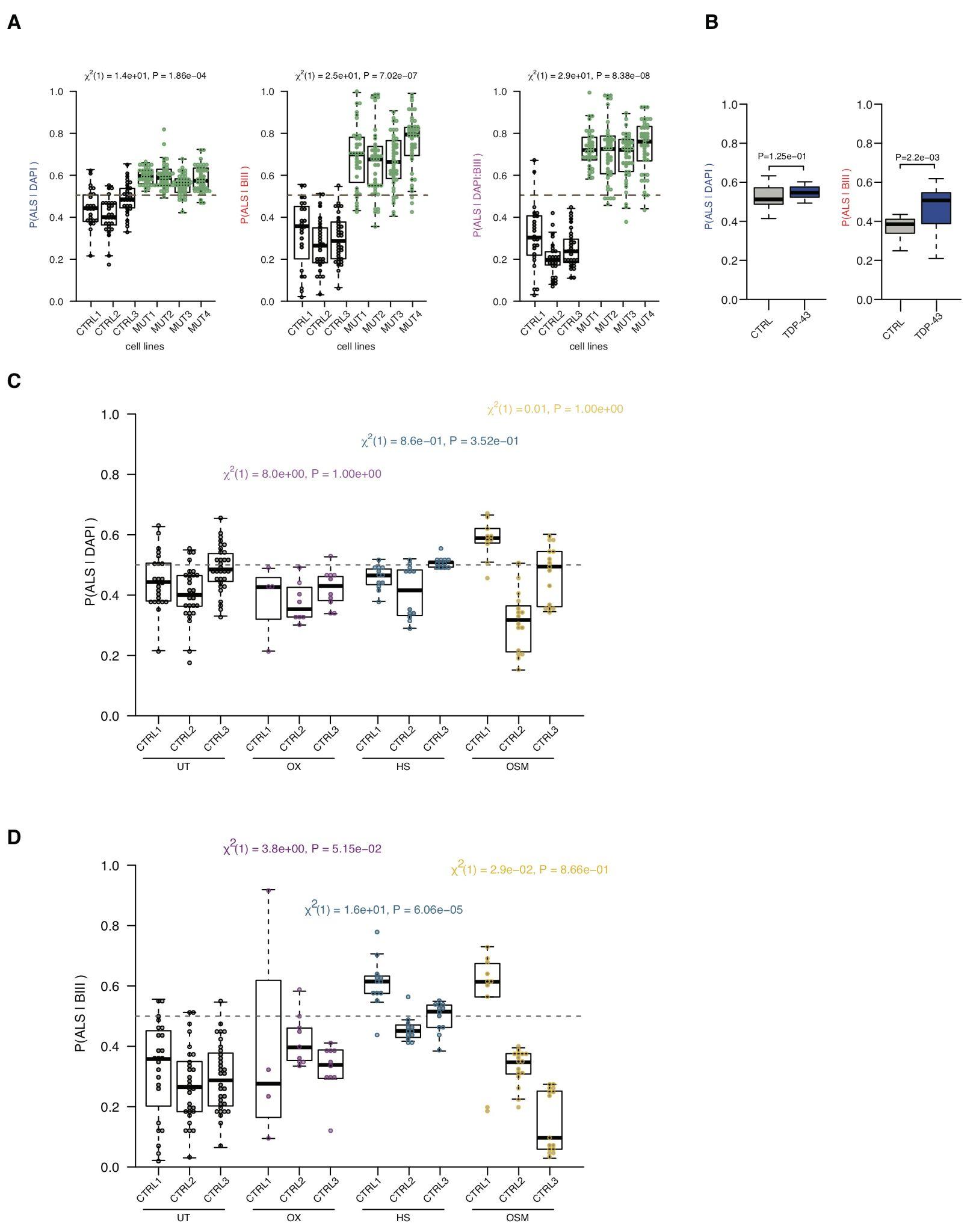


**Supplementary Figure 3 |** (**A**) Distributions of the *ALS|DAPI* (*left*), *ALS|BIII* (*centre*) and *ALS|DAPI:BIII* (*right*) model predictions for the individual MN cultures originating from 3 control cell lines (*grey dots*) and 4 VCP-mutant cell lines (*green dots*). Linear mixed effects analysis of the relationship between each model prediction and VCP mutation to account for idiosyncratic variation due to cell line or experiment differences. VCP mutation significantly increases *ALS|DAPI* predictions [$\chi^{2}\left( 1 \right)=14$ and $P=2.28e^{-04}$] by about $0.13\pm0.023$ (standard errors), *ALS|BIII* predictions [$\chi^{2}\left( 1 \right)=23$ and $P=2.1e^{-06}$] by about $0.4\pm0.033$ (standard errors), and *ALS|DAPI:BIII* predictions [$\chi^{2}\left( 1 \right)=29$ and $P=8.4e^{-08}$] by about $0.47\pm0.031$ (standard errors). (**B)** Distributions of the *ALS|DAPI* (*left*) and *ALS|BIII* (*right*) model predictions for the MN cultures originating from 3 control cell lines (*grey*) and 2 TARDBP-mutant lines (*blue*). Linear mixed effects analysis of the relationship between each model prediction and TDP43 mutation to account for idiosyncratic variation due to cell line or experiment differences. (**C**) Distributions of the *ALS|DAPI* model predictions for the individual MN cultures originating from three control cell lines after one hour of oxidative (magenta), heat (blue) and osmotic (yellow) stress. Linear mixed effects analysis of the relationship between each model prediction and individual treatment effect to account for idiosyncratic variation due to cell line or experiment differences. (**D**) Distributions of the *ALS|BIII* model predictions for the individual MNs cultures originating from 3 control cell lines after one hour of oxidative (magenta), heat (blue) and osmotic (yellow) stress. Linear mixed effects analysis of the relationship between each model prediction and individual treatment effect to account for idiosyncratic variation due to cell line or experiment differences.


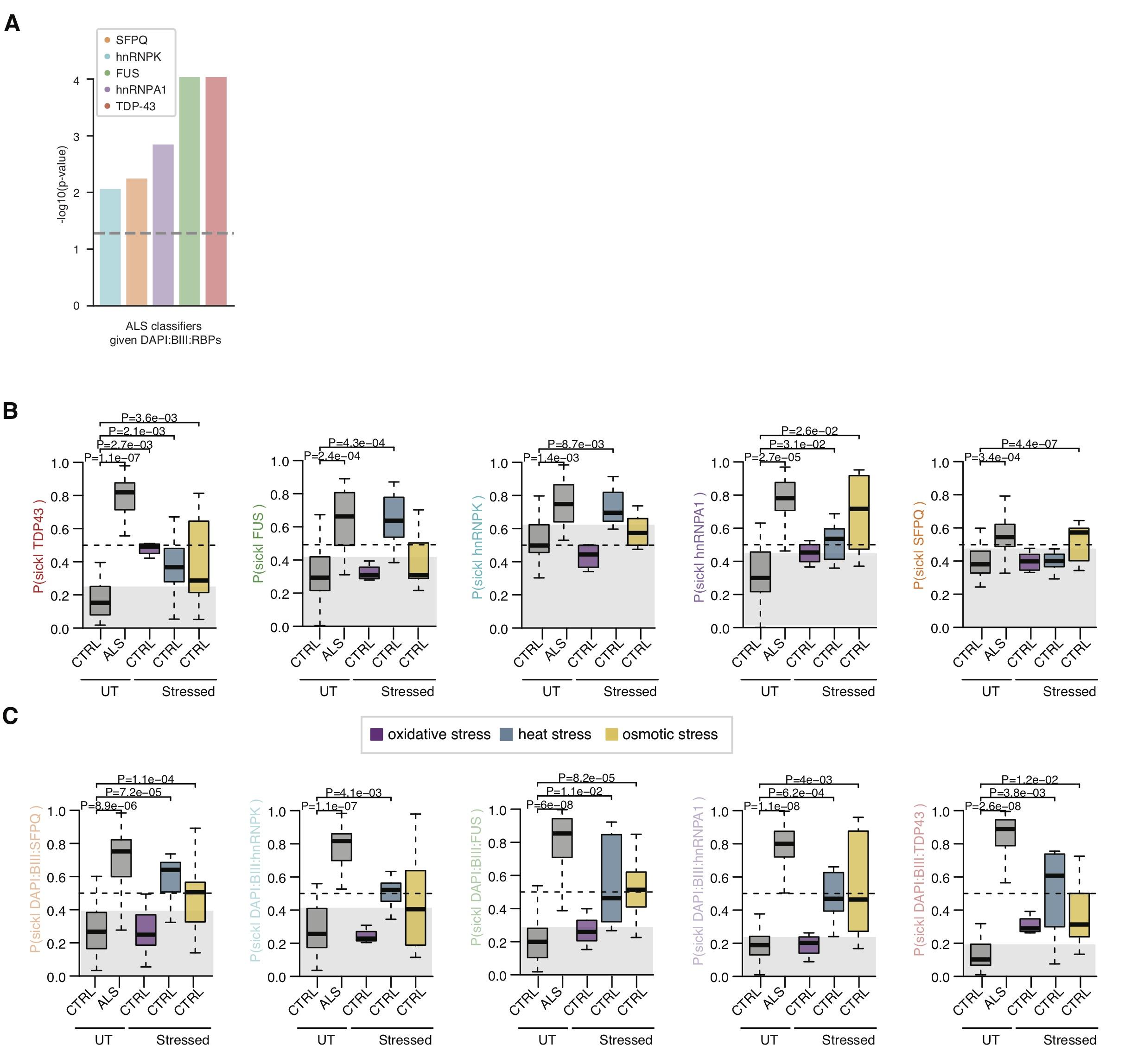


**Supplementary Figure 4 |** (**A**) Bar graphs representing the increase in performance as obtained from -log10(P-values) of one-sided Mann-Whitney test comparing the AUCs from the *ALS|DAPI:BIII* classifier and the AUCs from individual *ALS|DAPI:BIII:RBP* classifiers. (**B**) Boxplots showing the distributions *ALS|*RBPs model predictions on untreated control and ALS MN cultures, and control MN cultures after one hour of oxidative, heat and osmotic stress. Magenta = oxidative stress. Blue = heat stress. Yellow = osmotic stress. Stress treatment effect analysis on model prediction obtained using linear mixed effects analysis. P-values obtained from linear mixed models are indicated when significant. (**C**) Same as (B) for *ALS|DAPI:BIII:*RBPs model predictions.

**Electronic supplementary material**

Supplementary Tables 1-7 can be accessed [here](https://docs.google.com/spreadsheets/d/1wzDe6OO0dO_kJ8w-Wwd7EX_KnUVh1YMk4PSfck31DmY/edit?usp=sharing).

Table S1 | Description of human sample origin and mutations.

Table S2 | List of the ALS and stress trained CNN-based classifiers.

Table S3 | Performances of the 52 trained classifiers across the 10 folds.

Table S4 | ALS classifier assigned class probabilities for all the views from a cell culture (~ 10 per cell culture) that are then averaged to obtain a single per-culture classification probability.

Table S5 | Same as Table S4 for oxidative stress classifier.

Table S6 | Same as Table S4 for heat stress classifier.

Table S7 | Same as Table S4 for osmotic stress classifier.
